# Supplementary material for: Comparing corporate sustainability programmes, social entrepreneurship, and cooperatives in shaping farmers’ well-being
Source: Commun Sustain. 2026 Jan 23;1(1):20. doi: 10.1038/s44458-025-00020-8 (PMC12863647; doi:10.1038/s44458-025-00020-8)
Supplement: Supplementary file 2 — Supplementary Information [file 44458_2025_20_MOESM2_ESM.pdf]

Comparing corporate sustainability programmes, social entrepreneurship, and cooperatives in shaping farmers' well-being

(COMMSSUSTAIN-25-0116A)

Supplementary Information

Javier G. Montoya-Zumaeta, Christoph Oberlack, Ronja Barelli, Samuel Bruelisauer, Diego P. Zavaleta

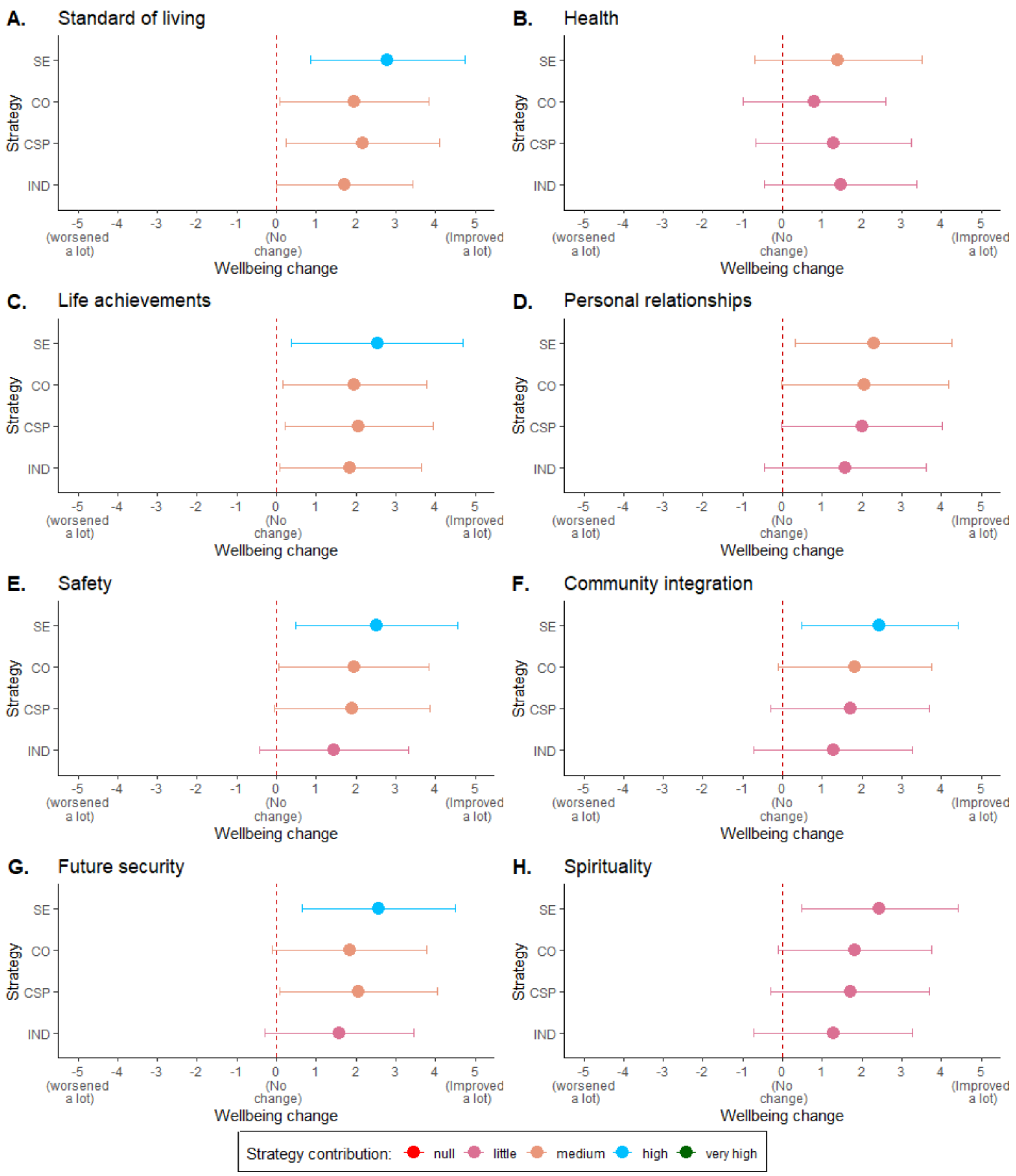

Fig. S1: Contribution of sustainability strategies to individual PWI components. Farmers self-reported changes in individual PWI-8 domains and the perceived contributions of sustainability strategies to these changes were collected using eleven- and five-point scales, respectively; and then averaged at the group-level to enable the comparisons shown here. We recorded null contribution when the group-level average of the perceived (sustainability strategy) contribution was between 0 and 1; little when it was between 1 and 2; medium when it was between 2 and 3; high when it was between 3 and 4; and very high when it was between 4 and 5. The groups refer to farmer engagement in social enterprises (SE), cooperatives (CO), corporate programmes (CSP), or no strategy (IND).

Table S1A. Descriptive statistics across farmer groups – based on strategies engaged in

	Strategies					
	**IND**	**CSP**	**CO**	**SE**	**All sample**	**Test**
Well-being index (PWI-8/100)	0.782 (0.126)	0.833 (0.124)	0.851 (0.099)	0.846 (0.111)	0.833 (0.116)	**<0.001**
Weights for the PWI-8:						
Material facilities	1.213 (0.665)	1.354 (1.392)	1.159 (1.238)	1.234 (1.538)	1.254 (1.282)	0.417
Health	2.315 (1.628)	2.685 (2.225)	2.416 (1.901)	2.182 (1.804)	2.481 (1.997)	0.156
Life achievements	1.112 (0.698)	1.024 (0.852)	0.930 (0.738)	1.065 (0.817)	1.009 (0.791)	0.248
Pers. relationships	1.258 (1.257)	1.358 (1.289)	1.346 (1.180)	1.558 (1.535)	1.364 (1.281)	0.490
Safety	1.034 (0.593)	0.882 (1.045)	0.780 (0.681)	0.805 (0.629)	0.860 (0.834)	**0.097**
Comm. integration	0.798 (0.457)	0.740 (0.746)	0.715 (0.604)	0.779 (0.620)	0.744 (0.649)	0.736
Future security	0.876 (0.540)	0.748 (0.795)	0.790 (0.743)	0.818 (0.739)	0.789 (0.739)	0.544
Spirituality	1.393 (1.557)	1.209 (1.392)	1.864 (1.956)	1.558 (1.916)	1.498 (1.709)	**<0.001**
Main product certified (Yes=1)	0.146 (0.355)	0.476 (0.500)	0.939 (0.239)	0.468 (0.502)	0.585 (0.493)	**<0.001**
Main product area (ha)	2.543 (3.394)	2.867 (2.265)	2.506 (1.716)	2.073 (1.437)	2.603 (2.226)	**0.038**
Income from main product (PEN)	59,314.225 (370,078.839)	26,969.016 (35,008.789)	21,348.084 (23,345.355)	21,854.675 (65,497.704)	28,991.161 (142,776.758)	0.182
Productivity (in PEN per ha)	10,077.532 (18,063.983)	9,498.451 (7,587.377)	8,406.356 (6,764.730)	8,575.848 (17,413.446)	9,108.950 (10,918.160)	0.567
Cocoa is the main product (Yes=1)	0.315 (0.467)	0.720 (0.450)	0.453 (0.499)	0.961 (0.195)	0.603 (0.490)	**<0.001**
Reliance on the main product (0–1)	0.906 (0.214)	0.891 (0.225)	0.927 (0.185)	0.882 (0.267)	0.904 (0.216)	0.256
Gender (Female=1)	0.180 (0.386)	0.197 (0.398)	0.182 (0.387)	0.312 (0.466)	0.203 (0.403)	**0.088**
Age	48.27 (15.67)	51.69 (13.61)	50.57 (12.8)	49.10 (12.87)	50.52 (13.59)	0.161
Complete basic education (Yes=1)	0.539 (0.501)	0.382 (0.487)	0.369 (0.484)	0.421 (0.497)	0.404 (0.491)	**0.037**
Off-farm inc. over last year (Yes=1)	0.483 (0.503)	0.307 (0.462)	0.322 (0.469)	0.468 (0.502)	0.356 (0.479)	**0.003**
Work more than 5 y with your buyer (Yes=1)	0.371 (0.486)	0.209 (0.407)	0.565 (0.497)	0.416 (0.496)	0.377 (0.485)	**<0.001**
More than 50% of production sold to other buyers (Yes=1)	0.236 (0.427)	0.150 (0.357)	0.136 (0.343)	0.234 (0.426)	0.167 (0.373)	**0.055**
Site:						
San Martín	30 (33.7%)	156 (61.4%)	182 (85.0%)	53 (68.8%)	421 (66.4%)	**<0.001**
Quillabamba	28 (31.5%)	63 (24.8%)	32 (15.0%)	3 (3.9%)	126 (19.9%)	
Selva Central	31 (34.8%)	35 (13.8%)	0 (0.0%)	21 (27.3%)	87 (13.7%)	
N	89 (14.0%)	254 (40.1%)	214 (33.8%)	77 (12.1%)	634 (100.0%)	

Table S1B. Descriptive stats across farmer groups – based on managed land sizes

	Based on their managed land size			
	**< 3 ha**	**3–10 ha**	**> 10 ha**	**Test**
Well-being index (PWI-8/100)	0.836 (0.120)	0.837 (0.114)	0.820 (0.114)	0.368
Weights for the PWI-8:				
Material facilities	1.228 (1.060)	1.298 (1.492)	1.222 (1.271)	0.790
Health	2.764 (2.136)	2.429 (2.053)	1.992 (1.411)	**0.001**
Life achievements	1.034 (0.763)	1.020 (0.866)	0.937 (0.690)	0.503
Pers. relationships	1.274 (1.211)	1.339 (1.301)	1.603 (1.363)	**0.055**
Safety	0.848 (0.805)	0.812 (0.788)	0.976 (0.967)	0.192
Comm. integration	0.719 (0.543)	0.694 (0.601)	0.897 (0.884)	**0.012**
Future security	0.783 (0.690)	0.767 (0.839)	0.841 (0.625)	0.652
Spirituality	1.350 (1.568)	1.641 (1.957)	1.532 (1.440)	0.154
Main product certified (Yes=1)	0.544 (0.499)	0.637 (0.482)	0.571 (0.497)	**0.098**
Main product area (ha)	1.487 (0.748)	3.046 (1.674)	4.072 (3.644)	**<0.001**
Income from main product (PEN)	13,407.426 (13,083.326)	30,836.665 (51,231.166)	57,930.635 (310,462.194)	**0.015**
Productivity (in PEN per ha)	8,829.829 (7,903.620)	9,835.836 (12,881.364)	8,235.275 (12,027.029)	0.368
Cocoa is the main product (Yes=1)	0.578 (0.495)	0.592 (0.492)	0.675 (0.470)	0.173
Reliance on the main product (0–1)	0.925 (0.191)	0.902 (0.224)	0.863 (0.245)	**0.033**
Gender (Female=1)	0.213 (0.410)	0.196 (0.398)	0.198 (0.400)	0.882
Age	48.582 (13.357)	50.780 (13.511)	54.040 (13.564)	**<0.001**
Complete basic education (Yes=1)	0.395 (0.490)	0.388 (0.488)	0.456 (0.500)	0.418
Engagement in strat.:				
CSP	116 (44.1%)	104 (42.4%)	34 (27.0%)	**<0.001**
IND	46 (17.5%)	30 (12.2%)	13 (10.3%)	
CO	83 (31.6%)	87 (35.5%)	44 (34.9%)	
SE	18 (6.8%)	24 (9.8%)	35 (27.8%)	
Off-farm inc. over last year (Yes=1)	0.350 (0.478)	0.343 (0.476)	0.397 (0.491)	0.566
Work more than 5 y with your buyer (Yes=1)	0.297 (0.458)	0.400 (0.491)	0.500 (0.502)	**<0.001**
More than 50% of production sold to other buyers (Yes=1)	0.163 (0.371)	0.167 (0.374)	0.175 (0.381)	0.963
Site:				
San Martín	183 (69.6%)	162 (66.1%)	76 (60.3%)	0.075
Quillabamba	54 (20.5%)	48 (19.6%)	24 (19.0%)	
Selva Central	26 (9.9%)	35 (14.3%)	26 (20.6%)	
N	263 (41.5%)	245 (38.6%)	126 (19.9%)	

Table S1C. Descriptive stats across farmer groups – based on their main crop

	**Main crop**		
	**Coffee**	**Cocoa**	**Test**
Well-being index (PWI-8/100)	0.830 (0.114)	0.835 (0.118)	0.610
<u>Weights for the PWI-8:</u>			
Material facilities	1.230 (1.310)	1.270 (1.265)	0.705
Health	2.468 (2.196)	2.490 (1.857)	0.896
Life achievements	0.996 (0.749)	1.018 (0.818)	0.729
Pers. relationships	1.321 (1.245)	1.393 (1.305)	0.494
Safety	0.885 (0.865)	0.843 (0.814)	0.536
Comm. integration	0.742 (0.737)	0.746 (0.585)	0.939
Future security	0.746 (0.624)	0.817 (0.805)	0.239
Spirituality	1.611 (1.834)	1.424 (1.620)	0.178
Main product certified (Yes=1)	0.627 (0.485)	0.558 (0.497)	**0.083**
Main product area (ha)	2.334 (2.285)	2.781 (2.172)	**0.013**
Income from main product (PEN)	36,664.492 (222,843.622)	23,929.173 (32,992.804)	0.272
Productivity (in PEN per ha)	10,309.184 (14,447.255)	8,309.881 (7,642.889)	**0.026**
Reliance on the main product (0–1)	0.932 (0.158)	0.885 (0.246)	**0.007**
Gender (Female=1)	0.171 (0.377)	0.225 (0.418)	**0.096**
Age	48.302 (14.019)	51.976 (13.108)	**<0.001**
Complete basic education (Yes=1)	0.381 (0.487)	0.420 (0.494)	0.329
<u>Engagement in strat.:</u>			
CSP	71 (28.2%)	183 (47.9%)	**<0.001**
IND	61 (24.2%)	28 (7.3%)	
CO	117 (46.4%)	97 (25.4%)	
SE	3 (1.2%)	74 (19.4%)	
Off-farm inc. over last year (Yes=1)	0.317 (0.466)	0.382 (0.487)	**0.096**
Work more than 5 y with your buyer (Yes=1)	0.337 (0.474)	0.403 (0.491)	**0.094**
More than 50% of production sold to other buyers (Yes=1)	0.226 (0.419)	0.128 (0.335)	**0.001**
<u>Site:</u>			
San Martín	170 (67.5%)	251 (65.7%)	**0.048**
Quillabamba	40 (15.9%)	86 (22.5%)	
Selva Central	42 (16.7%)	45 (11.8%)	
N	252 (39.7%)	382 (60.3%)	

Table S1D. Descriptive stats across farmer per sites

	Site			
	**San Martin**	**Quillabamba**	**Selva Central**	**Test**
Well-being index (PWI-8/100)	0.869 (0.102)	0.765 (0.119)	0.758 (0.097)	**<0.001**
<u>Weights for the PWI-8:</u>				
Material facilities	1.304 (1.495)	1.349 (0.624)	0.874 (0.661)	**0.011**
Health	2.641 (2.256)	2.198 (1.159)	2.115 (1.474)	**0.017**
Life achievements	0.967 (0.783)	1.111 (0.761)	1.069 (0.860)	0.149
Pers. relationships	1.254 (1.195)	1.254 (0.632)	2.057 (1.990)	**<0.001**
Safety	0.722 (0.797)	1.167 (0.576)	1.080 (1.123)	**<0.001**
Comm. integration	0.679 (0.632)	0.865 (0.407)	0.885 (0.920)	**0.002**
Future security	0.734 (0.825)	0.944 (0.478)	0.828 (0.554)	**0.017**
Spirituality	1.698 (1.998)	1.111 (0.596)	1.092 (0.948)	**<0.001**
Main product certified (Yes=1)	0.736 (0.441)	0.206 (0.406)	0.402 (0.493)	**<0.001**
Main product area (ha)	2.600 (2.033)	2.494 (1.815)	2.779 (3.386)	0.656
Income from main product (S/.)	26,910.508 (31,981.380)	13,078.921 (50,798.077)	62,104.920 (373,894.884)	**0.042**
Productivity (in S/. per Ha)	10,186.954 (7,667.576)	4,914.553 (12,936.226)	10,165.416 (17,788.264)	**<0.001**
Cocoa is the main product (Yes=1)	0.596 (0.491)	0.683 (0.467)	0.517 (0.503)	**0.048**
Reliance on the main product (0–1)	0.934 (0.178)	0.798 (0.288)	0.913 (0.219)	**<0.001**
Gender (Female=1)	0.164 (0.371)	0.325 (0.470)	0.218 (0.416)	**<0.001**
Age	48.44 (12.29)	57.29 (14.94)	50.78 (14.44)	**<0.001**
Complete basic education (Yes=1)	0.354 (0.479)	0.492 (0.502)	0.523 (0.502)	**0.001**
<u>Engagement in strat.:</u>				
CSP	156 (37.1%)	63 (50.0%)	35 (40.2%)	**<0.001**
IND	30 (7.1%)	28 (22.2%)	31 (35.6%)	
CO	182 (43.2%)	32 (25.4%)	0 (0.0%)	
SE	53 (12.6%)	3 (2.4%)	21 (24.1%)	
Off-farm inc. over last year (Yes=1)	0.375 (0.485)	0.310 (0.464)	0.333 (0.474)	0.357
Work more than 5 y with your buyer (Yes=1)	0.406 (0.492)	0.341 (0.476)	0.287 (0.455)	**0.075**
More than 50% of production sold to other buyers (Yes=1)	0.207 (0.405)	0.056 (0.230)	0.138 (0.347)	**<0.001**
N	421 (66.4%)	126 (19.9%)	87 (13.7%)	

Table S2. Average marginal effects from regressions – complete results

Variable	All sample	Farmer type based on managed land sizes			Main crop	
		< 3 ha	3–10 Ha	> 10 ha	Cocoa	Coffee
**Strategies:**						
CSP	0.0138	0.0134	0.0125	0.00907	-0.0178	0.0168
CO	0.00161	0.00728	-0.0052	-0.0082	-0.0205	0.00128
SE	0.0441***	0.0480**	0.0529**	0.0336*	0.0262**	0.0491*
**Site:**						
Quillabamba	-0.0581**	-0.0930***	-0.0404	-0.0128	-0.0628*	-0.0398**
Selva Central	-0.0954***	-0.118***	-0.0769***	-0.110***	-0.111***	-0.0821***
**Farmer type:**						
Medium (3-10 ha)	-0.00907				-0.00528	-0.0241
Large (>10 ha)	-0.0223*				-0.033***	-0.0171
**Household-level features:**						
Coffee/cocoa certified	0.0191	0.0066	0.0224	0.0258	-0.00267	0.0434**
Main product (1=cocoa)	0.000123	0.00482	0.00626	-0.00961		
Gross income from main product - PEN (ln)	0.00707	0.0146*	0.00167	0.0118	0.00795	0.0123
Main crop extension (ha)	0.00623**	-0.00436	0.0122***	0.00143	0.00574**	0.00681
More than half prod. sold to other buyers (Yes=1)	-0.0394***	-0.0359*	-0.0576***	-0.0226	-0.0550***	-0.0141
Hh members older than 16-y	-0.00228	0.00263	-0.00011	-0.0151	0.00567	-0.0170**
Gender (female=1)	-0.0462***	-0.0405**	-0.0559***	-0.0489*	-0.054***	-0.0459**
Age (in yrs-old)	-0.00161***	-0.00126**	-0.00218***	-0.00165	-0.002***	-0.00183**
Complete basic education (Yes=1)	-0.0102	-0.0237**	-0.00845	0.00469	-0.01	-0.0180*
N	599	249	236	114	243	356

*** p<0.01, ** p<0.05, * p<0.1

Table S3. Coefficients from the extended model including interactions between strategies and demographic characteristics

Variable	All sample
**Strategies:**	
CSP	0.103
CO	-0.00613
SE	0.509***
**Site:**	
Quillabamba	-0.482***
Selva Central	-0.683***
**Farmer type:**	
Medium (3–10 ha)	-0.076
Large (>10 ha)	-0.200**
**Household-level features:**	
Coffee/cocoa certified	0.111
Main product (1=cocoa)	-0.0117
Gross income from main product - PEN (ln)	0.0678
Main crop extension (ha)	0.0465**
More than half prod. sold to other buyers (Yes=1)	-0.297***
Hh members older than 16 y	-0.0262
Gender (female=1)	-0.183
Older than 60 y (yes=1)	-0.314***
Complete basic education (yes=1)	-0.0178
**Interactions:**	
CSP x Female	-0.248
CO x Female	-0.0242
SE x Female	-0.338
CSP x Older_60	0.136
CO x Older_60	0.075
SE x Older_60	-0.201
Constant	1.266***
N	599

*** p<0.01, ** p<0.05, * p<0.1

Table S4. Overview of organizations included in the analyses

#	Strategy case / site*	Scope of operations	Main economic activities	# affiliated and surveyed farmers	Certifications	Targeting	Organization structure	Funding sources next to cocoa/coffee revenues
1	Cooperative A / SM	Regional	• Export of cacao beans.<br>• Regional sales of their own branded chocolate.	~ 2000 (40)	Organic (Bio Suisse), Fairtrade, Biodynamic	• Initially, the cooperative targeted *cocaleros* farmers settled across its intervention area.<br>• Currently, they attempt to focus its field interventions on farmers willing to adopt organic practices.	• Farmers are grouped based on their closeness around several Comités comprised by 30 farmers as minimum within the intervention area of the organization.<br>• Each Comité select representatives for participating in the decision-making platform	• Competitive grants called by the government and external development agencies.<br>• Collaborations with environmental NGOs
2	Cooperative B / SM	Regional	• Export of coffee green beans.<br>• Sales of their own branded coffee locally and in Lima.	176 (30)	Organic, Rainforest, 4C, Starbucks C.A.F.E.	• Coffee farmers in the Moyobamba province growing high-quality coffee varieties.	• General Assembly celebrated twice per year.	• Competitive grants called by the government and external development agencies.
3	Cooperative C / SM	Regional	• Export of coffee green beans<br>• Local sales of their own branded coffee.	517 (30)	Organic, Fairtrade, Rainforest Alliance, Starbuck C.A.F.E.	• Coffee farmers in the Moyobamba province growing high-quality coffee varieties.<br>• Female coffee farmers (~30% of its associates)	• Farmers grouped in Comités.<br>• Each Comité select representatives for participating in the decision-making platform	• Competitive grants called by the national and local governments.
4	Cooperative D / SM	Regional	• Export of coffee green beans<br>• Sales of their own branded coffee locally, in	385 (30)	Organic, Fairtrade, Rainforest Alliance	• Coffee farmers settled around a protected area at northern of San Martin.	• Farmers grouped in Comités.<br>• Each Comité select representatives for	• Competitive grants called by the government and external development agencies.

			Lima and abroad (sporadically).				participating in the decision-making platform	• Collaborations with environmental NGOs
5	Social Enterprise A / SM	Regional	• Sales of their own branded chocolate locally and in Lima.	30 (12)	None	• Cocoa farmers residing in two indigenous communities settled around a protected area at northern San Martin	• Decisions are discussed and made into the frame of existing communal platforms.	• Competitive grants called by the government and external development agencies.<br>• Collaborations with environmental NGO
6	Social Enterprise B / SM	Regional	• Sales of their own branded chocolate mainly abroad.	32 (29)	None	• Cocoa farmers settled around a protected area of San Martin	• All farmers live in two towns close to each other facilitating communications among them<br>• Farmer's direct participation in defining the cocoa year price used by the enterprise.	• Own nonprofit foundation<br>• Competitive grants called by the government and external development agencies.<br>• Collaborations with environmental NGO
7	Cooperative E / SM	Regional	• Export of cacao beans.<br>• Regional sales of their own branded chocolates.	400 (40)	Organic, Fairtrade, Rainforest Alliance	• Initially, the cooperative targeted *cocaleros* farmers settled across its intervention area.<br>• Cocoa farmers around a protected area in San Martin.	• Farmers are grouped in several Comités comprised by 30 farmers as minimum.<br>• Each Comité select representatives for participating in the decision-making platform	• Competitive grants called by the government and external development agencies.<br>• Collaborations with environmental NGOs
8	Cooperative F / QU	Regional	• Export of coffee green beans<br>• Local sales of their own branded coffee and chocolate.	152 (25)	Fairtrade, USDA NOP (Organic USA).	• Farmers growing fine varieties of coffee and cocoa around Quillabamba.	• Farmers grouped in Comités.<br>• Each Comité select representatives for participating in the decision-making platform	• Competitive grants called by the government and external development agencies.

9	Company A / SM	Transnational	• Export of cocoa beans	2298 (40)	Fairtrade International, Fairtrade USA, Rainforest Alliance, EU Organic (Europe), USDA NOP (Organic USA), BioSuisse (CH), Fair for Life.	• Cocoa farmers across the most productive regions (San Martín, Huánuco, Ucayali and Junín) in Peru.	• Central collecting plant across different sites of Peru.<br>• Technical assistance offered to affiliated farmers.	• Direct funding from private companies to provide extension services to farmers.
10	Social Enterprise C – QU	National	• Coffee shop located in Cusco sourcing special coffees from the region	6 (3)	Kunan (Social Enterprise Network - Peru)	• Female farmers growing fine varieties of coffee across Peru.<br>• Also, women are trained in coffee quality aspects and have the priority for being hired in the coffee shop.	• Technical assistance offered to affiliated farmers.	• Competitive grants called by the government and external development agencies.
11	Social Enterprise D – SC	Regional	• Local sales of their own branded chocolates.	~ 40 (12)	None	• Cocoa farmers residing in indigenous communities settled in the Selva Central site.	• Decisions are discussed and made into the frame of existing communal platforms.	• Collaborations with environmental government agencies and NGOs
12	Company B / QU	National	• Nationwide sales of their own branded chocolates.	400 (28)	None	• Farmers growing fine varieties of cacao beans across the Quillabamba site.	• Central post-harvesting plant in Quillabamba.<br>• For farmers living distant, two smaller collecting centres have been established.	• Collaborations with environmental government agencies and NGOs
13	Company C / SM, SC	National	• Export of chocolates and products derived from cocoa	~ 7000 (40)	EU Organic (Europe), USDA NOP (Organic USA), JAS (Organic Japan), MAFRA (Organic Korea), IBD Brazil, FairTrade	• Cocoa farmers across different sites in Peru.	• Several collecting plants distributed across Peru.<br>• Technical assistance offered to affiliated farmers.	• Direct funding from private companies to provide extension services to provider farmers.<br>• Collaborations with environmental

					International Fair Trade, UTZ.			government agencies and NGOs
14	Company D / SM	National	• Export of cacao beans.	~ 3500 (40)	Fairtrade; Organic; BioSuisse; Rainforest Alliance	• Cocoa farmers growing high-quality cocoa.	• A pilot Farmer Field School is being implemented in the San Martin site.<br>• Central collecting plant across different sites in Peru.<br>• Technical assistance offered to affiliated farmers.	• Direct funding from private companies to provide extension services to farmers.
15	Social Enterprise E / SM	National	• Local sales of their own branded chocolates.	20 (12)	None	• Female farmers growing cocoa in its intervention area.<br>• Also, women are trained in chocolate elaboration and have the priority for being hired in the shop owned by the organization.	• Technical assistance offered to associated farmers.	• Competitive grants called by the government and external development agencies.
16	Company E / SM	Regional	• Sales of their own branded coffee locally and nationwide.<br>• Coffee shop located in Moyobamba sourcing special coffees from the region	50 (30)	Organic	• Farmers growing fine varieties of coffee around Moyobamba.	• Technical assistance offered to affiliated farmers.	• Competitive grants called by the government and external development agencies.

17	Company F / SM, SC	Transnational	• Trading of coffee green beans sourced from diverse origins	~ 7000 (40)	Rainforest, FairTrade, Organic (EU, US, Japan, Brazil, Mexico), Starbucks C.A.F.E.	• Coffee farmers across different sites in Peru.	• Central collecting plant across different sites of Peru.<br>• Technical assistance offered to affiliated farmers.	• Direct funding from private companies to provide extension services to farmers.
18	Company G / QU	Regional	• Local sales of their own branded chocolates.<br>• Local shop in Quillabamba.	~ 35 (12)	None	• Farmers growing fine varieties of cocoa around Quillabamba.	• Central collecting plant, and technical assistance offered to affiliated farmers.	• Competitive grants called by the government and external development agencies.<br>• Own profits

(*) Site abbreviations: QU (Quillabamba), SC (Selva Central), SM (San Martín).

Table S5. Coffee and cocoa farmers' key productive features from nationwide statistics

VARIABLES	ENA 2023 (n=1297)*		Sampled farmers (n=634)	
	Cacao (n=767)	Coffee (n=1173)	Cacao (n=405)	Coffee (n=288)
**Production:**				
Total production (kg. per household)	1 434.12 (2 972.43)	1 685.82 (7 610.99)	1 503.99 (2 139.94)	1 578.75 (2 218.07)
Harvested area (ha)	2.18 (5.58)	2.29 (6.73)	2.35 (2.08)	2.30 (2.23)
**Land sizes:**				
Small (<3 ha)	33.46%		41.48%	
Medium (3–10 ha)	38.01%		38.64%	
Large (>10 ha)	28.53%		19.87%	
**Demographic features:**				
Household members	3.16 (1.74)		3.65 (1.75)	
Age	52.44 (13.9)		50.52 (13.59)	
Female farmer	16.65%		20.34%	
Complete basic education	30.22%		40.44%	

(*) Own estimations based on data extracted from the Agricultural National Survey 2023[1] corresponding to cocoa and coffee producers from the same departments included in our study sites (Cusco, Junín, Pasco, and San Martin).

Supplementary reference:

1. INEI. 2024. Encuesta Nacional Agropecuaria 2023. Instituto Nacional de Estadística e Informática, Lima, Peru. Consulted on 20.02.2025. Available on: https://datosabiertos.gob.pe/dataset/encuesta-nacional-agropecuaria-ena-2023-instituto-nacional-de-estad%C3%ADstica-e-inform%C3%A1tica-inei.
